# Recognizing the role of surgical oncology and cancer imaging in the multidisciplinary approach to cancer: an important area of future scholarly growth for *BMC Cancer*

**DOI:** 10.1186/1471-2407-13-355

**Published:** 2013-07-23

**Authors:** Stephen P Povoski, Nathan C Hall

**Affiliations:** 1 Division of Surgical Oncology, Department of Surgery, Arthur G. James Cancer Hospital and Richard J. Solove Research Institute and Comprehensive Cancer Center, The Ohio State University Wexner Medical Center, Columbus, OH 43210, USA; 2 Department of Radiology, The Ohio State University Wexner Medical Center, Columbus, OH 43210, USA

## 

Cancer, far more than any other disease entity, requires a multidisciplinary approach. This multidisciplinary approach to cancer involves the integration of treatment strategies specifically set forth by surgical oncologists, medical oncologists, and radiation oncologists [1-3]. While early stage solid malignancies are frequently treated successfully with surgical therapy alone, higher-stage disease generally requires integration of surgery along with adjuvant therapies that are administered by the medical oncologists and/or radiation oncologists, both in a standard postoperative fashion, as well as in a preoperative/neoadjuvant fashion. Treatment planning and treatment implementation by surgical oncologists, medical oncologists, and radiation oncologists rely heavily on various cancer imaging modalities [4-10]. Therefore, integral to each of these cancer subspecialists is the critical role played by the cancer imaging physician. Realistically, none of these cancer subspecialists can work in a vacuum, and such integration of services is essential for optimizing success of treatment, minimizing complications, and impacting positively on long-term outcome.

*BMC Cancer*, which was conceived in January 2001 [11,12], has made enormous efforts to consider articles addressing all aspects of cancer basic science and clinical research, including articles that address pathophysiology, prevention, diagnosis, and treatment. However, *BMC Cancer* also recognizes that while submissions concerning molecular and cellular biology, genetics, epidemiology, immunology, translational research, and clinical trials related to medical oncologists and radiation oncologists have been well-represented, to the contrary, submissions concerning areas of interest to surgical oncologists and cancer imaging physicians have been far underrepresented. Therefore, in an effort to help reach out to and expand the surgical oncology and cancer imaging readership, *BMC Cancer* is introducing a new sub-section within the current “Clinical oncology” section that will be entitled “Surgical oncology and imaging”. It is hopeful that this new sub-section will be an important area of future scholarly growth for *BMC Cancer*.

Within the past several years, increasing interest has developed in attempting to maximize the integration of cancer imaging into the surgical treatment schema for solid malignancies [13,14]. The development of such a multimodal approach has been the driving force of our own multidisciplinary cancer detection and therapy group at The Ohio State University [14]. It has been our ongoing contention that such a multimodal approach to cancer detection and surgical treatment is critical for diagnostic accuracy, surgical planning, intraoperative identification of all diseased tissues, guidance of surgical resection, and verification of the completeness of the surgical resection. Additionally, a multimodal approach to cancer detection and surgical treatment allows for complete integration and coordination of services provided by physicians involved in the surgical management of cancer patients, including surgical oncologists, cancer imaging physicians, and pathologists [14-18]. It is our hope that the efforts of *BMC Cancer* to reach out to the surgical oncology and cancer imaging readership will enable further development and refinement of such multimodal approaches to cancer detection and surgical treatment. Furthermore, with the ongoing development of more relevant target-specific and antigen-specific cancer detection/imaging agents, we envision that this concept of a multimodal approach will ultimately encompass preoperative and perioperative cancer imaging, intraoperative cancer detection and surgical resection, postoperative and long-term surveillance cancer imaging, and a wide variety of potential adjuvant target-specific and antigen-specific cancer therapies [19]. Such an integrated multimodal approach holds much promise for ultimately improving the care and long-term outcome of future cancer patients.

In summary, we enthusiastically welcome the efforts put forward by *BMC Cancer* in reaching out to the surgical oncology and cancer imaging readership by the introduction of the “Surgical oncology and imaging” sub-section. We envision this to become an important area of future scholarly growth for *BMC Cancer* and look forward to receiving submissions in this area.

## Pre-publication history

The pre-publication history for this paper can be accessed here:

http://www.biomedcentral.com/1471-2407/13/355/prepub
